# Physical activity pattern of patients with interstitial lung disease compared to patients with COPD: A propensity-matched study

**DOI:** 10.1371/journal.pone.0277973

**Published:** 2022-11-21

**Authors:** Sofie Breuls, Cintia Pereira de Araujo, Astrid Blondeel, Jonas Yserbyt, Wim Janssens, Wim Wuyts, Thierry Troosters, Heleen Demeyer

**Affiliations:** 1 Department of Rehabilitation Sciences, KU Leuven, Leuven, Belgium; 2 Programa de Pós-Graduação em Ciências da Reabilitação, Universidade Federal de Ciências da Saúde de Porto Alegre (UFCSPA), Porto Alegre, Brazil; 3 Clinical Department of Respiratory Diseases, University Hospitals Leuven, BREATHE, Department CHROMETA, KU Leuven, Leuven, Belgium; 4 Department of Rehabilitation Sciences, Ghent University, Ghent, Belgium; Universite de Toulon, FRANCE

## Abstract

**Introduction:**

Physical activity (PA) is reduced in patients with interstitial lung disease (ILD) and chronic obstructive pulmonary disease (COPD). Evidence about the PA pattern of patients with ILD is scarce. If PA of patients with ILD would be comparable to COPD, it is tempting to speculate that existing interventions focusing on enhancing PA could be as effective in ILD as already shown in COPD. Therefore, we aimed to compare PA and the correlates with PA in matched patients with ILD, COPD, and healthy subjects.

**Materials and methods:**

Patients with ILD (n = 45), COPD (n = 45) and healthy subjects (n = 30) were propensity matched. PA level, pattern, and PA correlations with lung function and physical performance (6-minute walking distance and quadriceps force) were compared between groups.

**Results:**

Daily number of steps was similar in both patient groups (mean±SE: 5631±459 for ILD, 5544±547 for COPD, p = 0.900), but significantly lower compared to healthy subjects (10031±536, p<0.001 for both). Mean intensity of PA tended to be lower in the ILD group (mean±SE metabolic equivalents of task per day: 1.41±0.04) compared to COPD (1.52±0.05, p = 0.074) and healthy individuals (1.67±0.04, p<0.001). The pattern of PA over one day was found to be similar between the three groups. Lastly, the correlation between PA and 6-minute walking distance was significantly weaker in patients with ILD compared to patients with COPD (respectively r = 0.348 and r = 0.739; p<0.05 for both).

**Conclusions:**

For a given functional reserve, patients with ILD perform an equal amount of steps but perform PA at lower intensity compared to patients with COPD. Both groups are less active compared to healthy control subjects. Functional exercise capacity was shown to be only moderately related to PA. This can potentially influence the effectiveness of PA interventions that can be expected.

## Introduction

Physical inactivity is a common feature of patients with chronic respiratory diseases [1–5]. An extended period of physical inactivity can result in adverse health effects and physical disability [6]. The physical activity (PA) level has been shown to be an important predictor for morbidity and mortality in patients with chronic respiratory diseases such as chronic obstructive pulmonary disease (COPD) [6] and, more recently, interstitial lung disease (ILD) [2, 3, 7].

In recent years, various researchers aimed to characterize PA in patients with COPD by investigating PA across the disease severity [8], detailing the ‘pattern and bouts’ of PA [9], analyzing the pattern of PA during the day [10–12] and investigating the relationship between PA and the risk of exacerbation, quality of life and mortality [13–15]. Currently, the focus in COPD is to investigate interventions that may increase PA [16, 17]. Still, information characterizing PA pattern and behavior in patients with ILD or interventions with a focus to improve PA in patients with ILD is lacking.

ILD is an overarching group of diseases including more than 200 different pulmonary disorders in which the lung parenchyma is affected by inflammation or fibrosis [18]. Patients with idiopathic pulmonary fibrosis (IPF) and sarcoidosis, types of ILD included in the available literature, present a reduced PA level compared to healthy matched control subjects [2, 3, 19] and the PA level of patients with IPF is associated with functional exercise capacity (as measured by a field walking test), dyspnea, fatigue and quality of life, even when adjusted for lung function impairment [20]. In line with findings in COPD, functional exercise capacity was shown to be the strongest predictor of PA in these patients [20].

Although similarities of PA characteristics in patients with COPD and ILD can be found in literature, comparisons of PA between these populations or other respiratory patient populations are scarce. PA has already been compared between patients with COPD, bronchiectasis, severe asthma and control subjects [21], but these groups were not clinically matched for factors known to be associated to PA. So, interpretation of the results and comparison between the groups had to be done with caution.

In order to develop targeted interventions to enhance PA in patients with ILD, it is important to understand PA behavior and pattern in ILD, as well as knowing the determinants of PA in ILD. When similarities with COPD are confirmed, it is tempting to speculate that interventions might be equally effective in ILD compared to COPD. However, the underlying pathophysiology in both diseases, e.g. the presence of hyperinflation and airway obstruction in COPD, compared to the presence of fibrosis of the lung in ILD, might affect PA behavior throughout the day as well as duration of physical activity bouts, differently.

Therefore, the current study aimed to compare the PA level (amount and intensity), PA distribution over the day, PA bout duration and the correlates with PA in patients with ILD and a group of patients with COPD (matched for age, gender, functional exercise capacity and season of assessment); and healthy controls (matched for age, gender and season of assessment). Because functional exercise capacity is known to have a stronger relation to physical activity as compared to lung function, patient groups were matched for functional exercise capacity. We hypothesize that the PA level and pattern will be similar in both disease populations, for a given functional exercise capacity.

## Materials and methods

### Patient population

The subjects included in the current study were assessed between 2006 and 2013 in previous studies performed by our research group [8, 11, 22–26] at the University Hospitals Leuven. All subjects provided written informed consent before the start of the data collection in the individual trials and the current retrospective analysis was approved by the ethical committee UZ/KU Leuven (S-62591). The present analyses only retrieved data collected at baseline of the previous studies.

Three groups were included: 1) patients with ILD, 2) patients with COPD, and 3) healthy control subjects. The ILD group presented a clinical diagnosis of ILD according to internationally established criteria with a formal workup and multidisciplinary discussion [27, 28], experienced dyspnea on exertion and were on stable medical therapy with no infection or exacerbation in the four weeks prior to the protocol. Patients with a diagnosis of Sarcoidosis were not included. The COPD group included patients aged between 40 and 80 years old, with a clinical diagnosis confirmed by post bronchodilator spirometry and also clinically stable (i.e. no medication or exacerbation) in the four weeks prior to the protocol. The healthy control group included subjects aged between 40 and 80 years old, with normal spirometry, never smokers or former smokers who did not develop airflow obstruction (FEV_1_/FVC <70%) in the further six years [25, 26].

Exclusion criteria for all groups [8, 11, 22–26] were inability to walk without walking aids, presence of significant co-morbid conditions or orthopaedical problems that would preclude the participants to be physically active, being diagnosed with psychiatric or cognitive disorders, progressive neurological or neuromuscular disorders and having nickel allergy (which precludes measurement of physical activity with the SenseWear device). Subjects who did not speak the Dutch language were also excluded. Specific exclusion criteria for patients with ILD were systemic manifestations not allowing training and a life expectance below three months [22]. Only patients with a valid physical activity measurement (see further) and available data on the six-minute walking distance (6MWD), gender and age were included in the propensity matching selection.

### Physical activity

**Fig 1:**
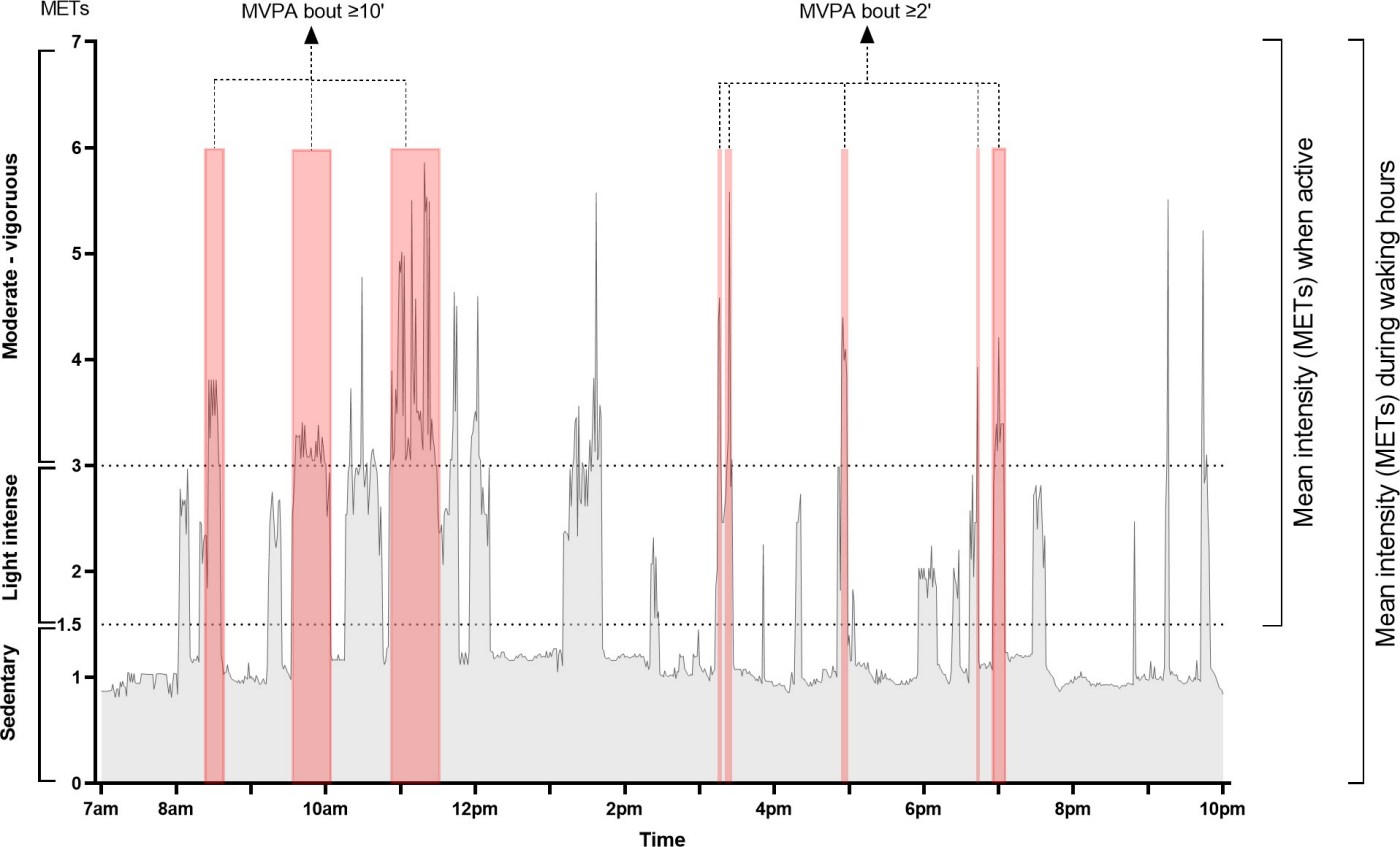
Example of a physical activity report of one day. Example of a physical activity report of one day, measured by SenseWear Pro armband. **Mean intensity (METs) during waking hours**: mean energy expenditure expressed in metabolic equivalent of a task between 7AM until 10PM. **Mean intensity (METs) when active**: mean energy expenditure expressed in METs when METs level of 1.5 is exceeded. **MVPA bout ≥2’**: period of at least two minutes when METs level ≥3. Total time MVPA per day is the summation of these periods in one day. MVPA bout ≥2’ is also used to calculate the mean duration of a MVPA bout. **MVPA bout ≥10’**: period of at least 10 minutes when METS level ≥3.

In all patients, PA was measured using the same activity monitor (SenseWear Pro armband, BodyMedia, Inc). Patients were instructed to wear the activity monitor for at least one week. Only patients with at least four valid days of PA measurement were included for the propensity matching selection. Days with at least eight hours of measurement, between 7AM and 10PM were considered as valid days [29]. The minute-by-minute output of each PA measurement including the number of steps and metabolic equivalents of task (METs) were exported for further analysis using the software SenseWear Professional 7.0 (BodyMedia, Inc). For all outcomes, data before 7AM and after 10PM were excluded [29]. As an indication of season, the duration of daylight on each measurement day was calculated [10]. For all outcomes, a mean of all valid days was calculated. Using the statistical software SAS 9.4, the following outcomes were calculated (Fig 1): 1) total amount of PA expressed as mean daily step count, 2) intensity of PA expressed as mean METs: A) during waking hours (7AM and 10PM) and B) when active (≥1.5 METs), 3) intensity based PA outcome: A) mean daily time in moderate to vigorous physical activity (MVPA) (METs ≥3.0). To exclude measurement errors, minutes of MVPA were included when they were performed for a minimum of two consecutive minutes (‘bouts’). And B) Sedentary time, expressed as percentage time of <1.5 METs of the wearing time, 4) pattern of PA focusing on bouts of MVPA: A) the mean duration of a MVPA bout per day, B) number of MVPA bouts ≥10 minutes per day, C) the percentage of the days of the measurement period with at least one MVPA bout ≥10 minutes, D) the percentage of patients without a MVPA bout ≥10 minutes during the measurement period, as a measure of the ability to perform a MVPA bout.

### Functional exercise capacity

Two 6MWD tests were conducted for each patient, to control for a potential learning effect [30]. The best test was taken for analyses. For patients (both COPD and ILD) who were on long-term oxygen therapy the usual flow was increased by one liter/minute to perform the 6MWD test. For the other patients with COPD, tests were conducted on room air. If a significant desaturation occurred during the first 6MWD test, the second was performed with oxygen supplementation. For the other patients with ILD, tests were conducted with an oxygen supplementation of two liters by default to avoid important desaturation [22].

### Other measurements

Data regarding age, gender, body mass index (BMI), lung function [forced vital capacity (FVC), forced expiratory volume in the first second (FEV_1_), functional residual capacity, total lung capacity (TLC), residual volume and diffusing capacity for carbon monoxide (DL_CO_)], and isometric quadriceps force (QF; Biodex® Medical Systems, USA) were retrieved from previous studies’ databases [8, 11, 22–26]. Since no general cutoff values exist to determine disease severity for patients with interstitial lung disease, we extended the GAP score (gender age and physiology score) for IPF to all patients with ILD [31]. Based on lung function parameters, patients with COPD were classified into GOLD categories (global initiative for chronic obstructive lung disease).

### Propensity matching

We retrieved from the previous trials databases 48 patients with ILD, 135 patients with COPD and 77 healthy subjects that fulfilled the study criteria. Subjects with ILD and COPD were selected using propensity matching by gender, age, mean daylight during the PA assessment and 6MWD. The healthy controls were selected using propensity matching by gender, age and mean daylight during the PA assessment in order to match the subjects with ILD already selected. The propensity matching was done using the SPSS 22.0 (IBM Corp., Armonk, NY, USA). All groups were matched with a matching score of 0.2.

### Data analysis

Data distribution was analyzed with the Shapiro-Wilk test and data are reported as mean ± standard error of the mean unless stated differently. First, total population, matched patients and unmatched patients with ILD characteristics and groups’ physical activity were compared using Generalized Linear Model. Second, to compare the PA pattern throughout the day, we plotted the active time and the percentage of active time (METs level ≥1.5) per group (i.e. ILD, COPD, healthy). Also, the coefficient of variance for daily steps was calculated for each individual patient as standard deviation/mean and compared between groups using Generalized Linear Model. Third, we investigated the relationship between physical activity (daily steps) and main clinical characteristics (i.e. age, BMI, percentage predicted values of lung function, functional exercise capacity and Quadriceps force [32]) with a Pearson test. To evaluate the possible influence of the type of ILD on the results, the association between daily steps and functional exercise capacity was compared within the ILD group between two ILD classes: patients with and without IPF. A correlation coefficient between 0.3–0.5 was considered a weak correlation, between 0.5–0.7 a moderate correlation, between 0.7–0.9 a strong correlation and higher than 0.9 a very strong correlation [33]. Correlations between groups were compared by examining the confidence intervals. For all analyses, the ILD group was taken as reference group, therefore comparisons are only reported between ILD and COPD, and between ILD and controls. The SAS 9.4 statistical software was used for all analyses and statistical significance was set at 5%. The plots were created using GraphPad Prism 8.

## Results

### Patient population

Out of 260 subjects in total, 120 were propensity matched (ILD n = 45, COPD n = 45, healthy n = 30). Age, gender and daylight were comparable between groups after matching, as was functional exercise capacity for both patient groups. Three patients with ILD were not included in the matched group because of the higher proportion of male patients and lower BMI (S1 Table). Other patients’ characteristics before and after propensity matching are described in Table 1. Hence, groups with matching age, gender, anthropometrics, daylight and functional exercise capacity were formed.

**Table 1:** Characteristics for ILD, COPD and healthy controls before and after propensity matching analysis.

Before matching													After matching										
		ILD (n = 48)			COPD (n = 135)			p-value	Healthy (n = 77)			p-value	ILD (n = 45)			COPD (n = 45)			p-value	Healthy (n = 30)			p-value
Age	years	66	±	1.3	65	±	0.6	0.915	61	±	0.7	**0.001**	66	±	1	65	±	1	0.445	63	±	1	0.164
Gender	M/F (%M)	31/17		(65%)	105/30		(77%)	0.084	47/30		(61%)	0.671	31/14		(69%)	29/16		(64%)	0.515	18/12		(60%)	0.438
BMI	kg/m^2^	27.5	±	0.7	26.6	±	0.5	0.278	25.8	±	0.4	0.063	27.8	±	0.7	27.1	±	0.8	0.438	26.1	±	0.6	0.123
6MWD	m	477	±	17	488	±	11	0.578	665	±	9	**< .0001**	480	±	18	497	±	18	0.474	655	±	12	**< .0001**
Daily steps	n/day	6274	±	486	5443	±	307	0.154	10480	±	378	**< .0001**	5631	±	459	5544	±	547	0.900	10031	±	536	**< .0001**
Daylight	min/day	815	±	22	713	±	16	**0.001**	692	±	21	**0.001**	811	±	23	778	±	29	0.283	753	±	34	0.146

Results shown as mean ± standard error of the mean. M/F: male/female; %M: percentage of males in the sample; BMI: body mass index; 6MWD: six-minute walking distance. P-value for comparison between ILD-COPD and ILD-healthy.

Of the 45 matched patients with ILD, 24 had idiopathic interstitial pneumonias, including 17 with IPF. Eleven patients had extrinsic allergic alveolitis, one drug induced ILD, one asbestosis and six connective tissue disease-associated interstitial lung disease. In two patients, no classification could be made (S2 Table). Twenty-five patients with ILD were categorized in GAP stage I, 15 in GAP stage II and five in GAP stage III. Of the 45 matched patients with COPD, 11 had mild COPD (GOLD I), 16 had moderate COPD (GOLD II), 16 had severe COPD (GOLD III) and two had very severe COPD (GOLD IV). As expected, patients with ILD had lower lung volumes (FVC and TLC) and patients with COPD had lower FEV_1_/FVC ratio (Table 2). Also, isometric quadriceps force was comparable between patient groups but was significantly lower compared with the control subjects (Table 2). Oxygen dependency was limited in both patient groups in daily life, but was higher in patients with ILD during the 6MWT.

**Table 2:** Patient characterization after propensity matching analysis.

		ILD (n = 45)			COPD (n = 45)			p-value	Healthy (n = 30)			p-value
**Age**	years	66	±	1	65	±	1	0.455	63	±	1	0.164
**Gender**	M/F (%M)	31/14		(69%)	29/16		(64%)	0.663	18/12		(60%)	0.438
**Height**	m	1.68	±	0.01	1.68	±	0.01	0.766	1.71	±	0.02	0.299
**Weight**	Kg	79	±	2	76	±	3	0.386	76	±	3	0.442
**BMI**	kg/m^2^	27.8	±	0.7	27.1	±	0.8	0.438	26.1	±	0.6	0.123
**FVC**	L	2.76	±	0.14	3.26	±	0.13	**0.009**	4.17	±	0.15	**< .0001**
**FVC**	% pred	81	±	3.4	97	±	3.3	**0.001**	119	±	2.7	**< .0001**
FEV_1_	L	2.15	±	0.08	1.59	±	0.09	**< .0001**	3.22	±	0.11	**< .0001**
FEV_1_	% pred	81	±	2.7	60	±	3.4	**< .0001**	115	±	3.1	**< .0001**
FEV_1_/FVC		80	±	1	49	±	2	**< .0001**	77	±	1	**0.259**
**FRC**	% pred	81	±	4.3	165	±	6.0	**< .0001**	118	±	3.6	**< .0001**
**TLC**	% pred	70	±	2.6	114	±	2.8	**< .0001**	105	±	1.8	**< .0001**
DL_CO_	% pred	44	±	2.0	59	±	3.5	**< .0001**	97	±	2.6	**< .0001**
**6MWD**	m	480	±	18	497	±	18	0.474	655	±	12	**< .0001**
**6MWD**	% pred	77	±	2.5	79	±	2.7	0.573	101	±	1.8	**< .0001**
**QF**	Nm	121	±	7.0	126	±	6.3	0.618	154	±	8.3	**0.002**
**QF**	% pred	79	±	4.3	85	±	3.8	0.304	102	±	4.0	**0.001**
O_2_ ADL	% patients	9			2			0.167	0			**0.001**
O_2_ 6MWT	% patients	100			9			< .0001	0			**0.001**

Results shown as mean ± standard error of the mean. M/F: male/female; %M: percentage of males in the sample; BMI: body mass index; FVC: forced vital capacity; FEV_1_: forced expiratory volume in the first second; FEV_1_/FVC: Tiffeneau index; FRC: functional residual capacity; TLC: total lung capacity; DL_CO_: diffusion capacity for carbon monoxide; 6MWD: six-minute walking distance; QF: quadriceps force; O_2_ ADL: oxygen supplementation during activities in daily life; O_2_ 6MWT: oxygen supplementation during six-minute walking test. P-value for comparison between ILD-COPD and ILD-healthy.

### Physical activity

The mean wearing period of the SenseWear in the matched patient groups was 8.2±0.2 days for ILD, 6.0±0.2 days for COPD and 6.7±0.1 days for healthy controls (both p<0.0001 compared to ILD). A significantly higher mean daily wearing time was observed in ILD compared to COPD (Table 3).

**Table 3:** Physical activity characteristics of patients with ILD, COPD and healthy controls.

	ILD (n = 45)			COPD (n = 45)			p-value	Healthy (n = 30)			p-value
Daily steps (n)	5631	±	459	5544	±	547	0. 900	10031	±	536	**< .0001**
Mean METs during waking hours*	1.41	±	0.04	1.52	±	0.05	0.074	1.67	±	0.04	**0.001**
Mean METs when active**	2.47	±	0.04	2.59	±	0.07	0.103	2.80	±	0.05	**< .0001**
Total time MVPA per day (min)	44	±	6	65	±	11	0.077	86	±	8	**0.002**
Mean duration MVPA bout (min)	4	±	0.2	4.5	±	0.3	0.114	5.7	±	0.4	**< .0001**
MVPA bouts per day (n)	3	±	0.4	3	±	0.4	0.543	4	±	0.3	**0.013**
% days with 10’ MVPA bout	36.6	±	4.8	42.6	±	5.7	0.386	66.0	±	4.6	**0.001**
% patients with at least one 10’ MVPA bout during the measurement period	73			67			0.490	100			**0.002**
Sedentary time (%)	70	±	2	68	±	2	0.224	62	±	2	**0.001**
Wearing time (min)	878	±	4	837	±	10	**0.001**	876	±	6	0.825

Results shown as mean ± standard error of the mean. METs: Metabolic equivalent of a task

*Mean METs during waking hours between 7AM and 10PM

**Mean METs when active: when METS level is ≥1.5; MVPA: moderate to vigorous physical activity when METs ≥3.0. Sedentary time: % of wearing time when METs level <1.5. P-value for comparison between ILD-COPD and ILD-healthy.

Patients with ILD accumulated significantly less steps per day when compared to the steps of the healthy controls, but this was not different from patients with COPD (Table 3). The mean PA intensity per day tended to be lower in patients with ILD compared to patients with COPD and was significantly lower compared to healthy controls, both for mean intensity during waking hours as mean intensity during activity. Daily time spent in MVPA tended to be lower in patients with ILD compared to patients with COPD and was lower in patients with ILD compared to healthy controls. Roughly 70% of the patients with ILD and COPD achieved at least once an MVPA bout of minimum 10 minutes during the measurement period, compared to 100% of the healthy controls. Such a bout was achieved on 66% of the days in the healthy controls compared to 37% (ILD) and 43% (COPD) of the days in the patient groups.

**Fig 2:**
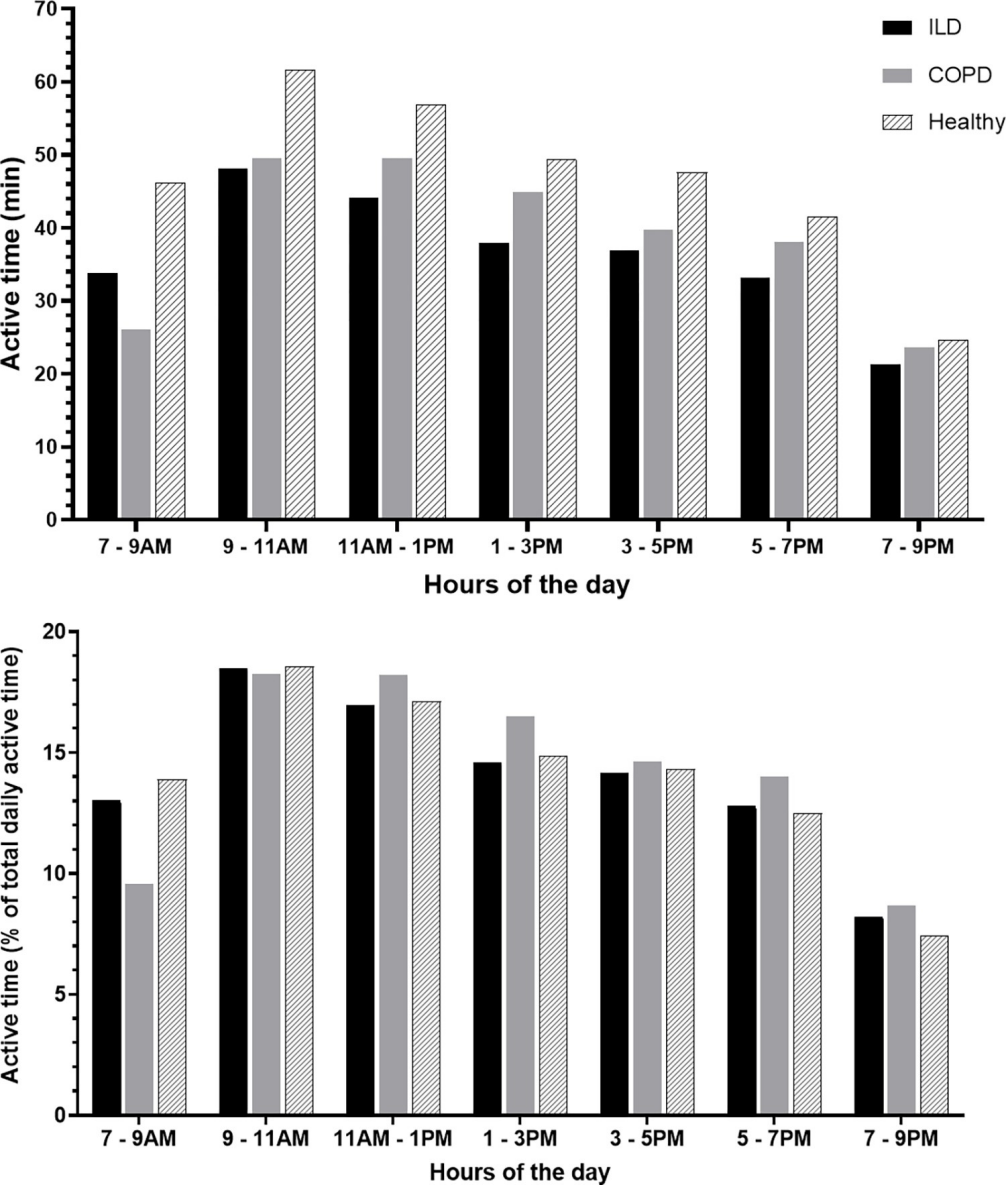
Absolute and relative active time per day in patients with ILD, COPD and healthy controls. Distribution of active periods (METs level ≥1.5) in a day, expressed as the amount of active minutes per two hours (panel A) and the relative distribution of a patient’s active time over the day during waking hours (7AM to 9PM) (panel B) in patients with ILD, COPD and healthy controls.

Fig 2 depicts the distribution of active time over the day, given in absolute (panel A) and relative active time (panel B). A comparable pattern is observed between all the groups, except for patients with COPD with less active time between 7 and 9AM. The coefficient of variation of daily steps was 0.35, 0.37 and 0.38 for ILD, COPD and the healthy group respectively and was found not to be significantly different between groups.

### Relationship between physical activity and clinical characteristics

**Fig 3:**
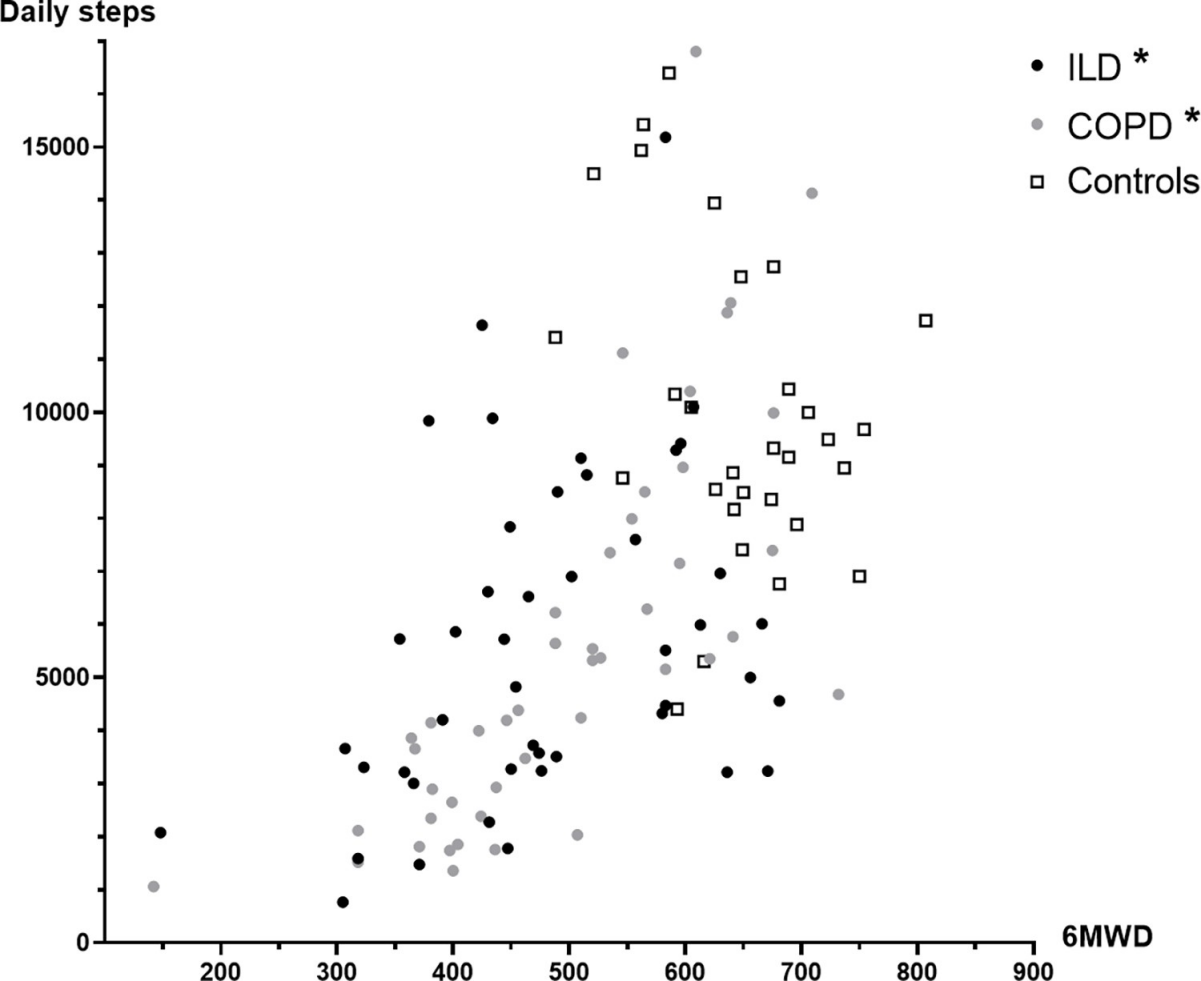
Correlation between functional exercise capacity and physical activity in patients with ILD, COPD and healthy controls. Correlation between six-minute walking distance (x-axis) and daily steps (y-axis) in patients with ILD (black dots), COPD (grey dots) and healthy controls (open squares). *P<0.05.

Correlations between daily steps and patient characteristics are provided in the online supplement (S3 Table). The correlation between PA and 6MWD was weaker in patients with ILD compared to patients with COPD (Pearson r = 0.348 for ILD and r = 0.739 for COPD) (Fig 3). Within the ILD group, the correlation between PA and 6MWD was comparable across the different ILD classes (Pearson r in IPF group r = 0.30 and non-IPF group r = 0.39). In ILD, no correlation was found between PA and lung function parameters (r = 0.170 for FVC %pred and r = 0.228 for DL_CO_ %pred), whereas in COPD, a weak to moderate correlation between PA and lung function parameters (r = 0.524 for FEV_1_%pred and r = 0.554 for DL_CO_ %pred) was observed. In both patient groups, no correlation was found between PA and isometric quadriceps force. In the healthy group, daily steps showed no significant correlation with any of the included characteristics (Fig 3).

## Discussion

This paper provides a detailed description of the PA levels and profiles throughout the day of patients with ILD compared to patients with COPD matched for anthropometric characteristics and functional exercise capacity and as well as to healthy age matched control subjects. In summary, we found 1) similar reductions in the number of steps per day, but PA intensity was lower in patients with ILD compared to COPD and 2) functional exercise capacity, known as a strong correlate of PA in COPD, was only weakly related to PA in patients with ILD.

### Comparison with previous findings

This study showed a lower amount and intensity of PA in patients with ILD and COPD compared to healthy controls. This corroborates the findings of previous work in this field [1, 2]. With a mean daily step count of less than 6000 for ILD and COPD, the recommended levels of PA (~7000–8000 steps/day) to maintain health were not achieved [34]. Patients in the ILD group performed PA at lower intensity than patients with COPD, although both groups were matched based on functional exercise capacity (6MWD). We can speculate that dyspnea and severity symptoms during activity may be different between groups that caused patients with ILD to perform physical activity at a less intense level. Perhaps the 6MWD was slightly overestimated in the patients with ILD, as all patients conducted the test with oxygen supplementation. Others have reported a consistent acute improvement of the 6MWD on oxygen supplementation in patients with ILD [35, 36]. This could explain why for a given 6MWD, the PA intensity in daily life was lower. Unfortunately, we did not have 6MWD tests available in these patients without oxygen supplementation.

Despite this observation, recent literature in the healthy population showed that, to reduce all-cause mortality, the step-mortality association was found to be stronger than the intensity-mortality association in US adults [37]. Therefore, it might be more important to focus on the total volume of PA expressed in number of steps per day.

An in-depth analysis on the PA pattern in terms of MVPA bouts showed the difficulty of both patient groups to accomplish bouts of moderate to vigorous intense exercise. Both patients with ILD and COPD take shorter activity bouts, when doing activities with at least moderate intensity. Around 1/3 of patients did not even perform a single MVPA bout of 10 minutes during the seven days assessment period. However, the health consequences of lack of moderate-to-high intensity exercise are up for debate, as it was recently shown that reduction in mortality risk is independent of how moderate-to-high intensity activity is performed, in bouts or dispersed [38].

The distribution of PA over the day appeared similar in patients of both diseases and is in line with the PA pattern previously published in patients with COPD, in different countries [29]. Patients were most active around noon, followed by a decrease in activity towards the evening [12].

In 2005, Pitta et al. were the first to investigate the relationship between PA and physical functions in patients with COPD [1]. Daily walking time was found to be strongly and positively related to functional exercise capacity. This relation was also observed in other patients with COPD [6] and in other obstructive airway diseases, e.g. severe asthma and bronchiectasis [21]. This observation cannot be extended to patients with ILD based on the present study as the correlation between PA and functional exercise capacity in our results was found to be weaker. This is in line with previous research in patients with fibrotic idiopathic interstitial pneumonia [2], but others have shown better associations in patients with IPF [20] and in patients with ILD waiting for lung transplantation (Pearson r = 0.59, p<0.01) [39]. However, in the latter study, PA measurement included rehabilitation sessions, and is thus not presenting unsupervised physical activity in daily living. The reason for the weaker correlation in patients with ILD compared to COPD is not very clear and more data are needed. A possible explanation might be the heterogeneity in pathologies in ILD. In addition, other unmeasured symptoms (such as chronic cough) may be present, or to a greater extent, in patients with ILD which may have an impact on the PA level. However, these are speculations and should be further investigated. Given this weak correlation between PA and functional exercise capacity, estimates of PA based on functional exercise capacity are not recommended. Instead, the assessment of objective PA becomes even more important in patients with ILD as in COPD, reflective of the overall worse prognosis in ILD compared to COPD.

### Strengths and limitations

This study is, to the best of our knowledge, unique in its design by matching two patient populations with a healthy control group and comparing daily physical activity. To this end, we propensity matched patients with a larger pool of collected data. The three groups were matched for daylight, to reduce the possible impact of seasonal variations on PA [10]. PA was objectively measured in the three groups using the same activity monitor (SenseWear Pro armband, BodyMedia, Inc), a valid device for measuring PA in patients with COPD [40]. In all groups, the PA measurement was performed for a minimum of six days, fulfilling the recently published recommendations [29]. Also, the coefficient of variation showed that the three groups had a similar extent of variability in their PA. This implies that day-to-day variability is comparable and collectively suggest that recent guidelines on how to assess and process physical activity in COPD [29] probably can also adopted in ILD.

Nevertheless, the following weaknesses should be taken into account when interpreting the data. First, data was collected in the period between 2009 and 2013. However, all data were analyzed with the most recent software, considering the current post processing guidelines [29]. Second, since we know the prognosis of IPF is worst of all ILD diagnoses, PA outcomes may differ between patients with IPF and non-IPF. However our sample size was too small to explore this more thoroughly.

### Practical implications and future

From the results of this study, we can infer that identification of inactivity in patients with ILD requires specific attention and should not be inferred from functional assessment including walking tests. Management of symptoms and the behavioral component in PA enhancing interventions may become more important. These behavioral interventions, effective in patients with COPD [6], could also offer positive outcomes in PA in patients with ILD.

## Conclusions

This study showed that patients with ILD and COPD experience similar PA reductions in terms of total PA compared to that of healthy control subjects, although patients with ILD perform PA at a lower intensity. PA in ILD seems also less dependent of individual functional capacity and lung function impairment. This may have consequences in the implementation of PA enhancing programs.

## Supporting information

S1 Table Characteristics for ILD group for matched and unmatched patients. Results shown as mean ± standard error of the mean. M/F: male/female; %M: percentage of males in the sample; BMI: body mass index; 6MWD: six-minute walking distance. P-value for comparison between ILD matched and unmatched population.
(DOCX)

S2 Table Diagnosis of ILD patients, n (%). IIP: idiopathic interstitial pneumonias; IPF: idiopathic pulmonary fibrosis; INSIP: idiopathic nonspecific interstitial pneumonia; COP: cryptogenic organizing pneumonia; IIP: idiopathic interstitial pneumonias; EAA: extrinsic allergic alveolitis; CTD-ILD: connective tissue disease-associated interstitial lung disease.
(DOCX)

S3 Table Correlations between daily steps and clinical characteristics. BMI: body mass index; FEV1: forced expiratory volume in the first second; FVC: forced vital capacity; DLCO: diffusion capacity for carbon monoxide; 6MWD: six-minute walking distance; QF: quadriceps force. r: Pearson correlation coefficient; p: p-value.
(DOCX)
