# Supplementary material for: Physical activity pattern of patients with interstitial lung disease compared to patients with COPD: A propensity-matched study
Source: PLoS One. 2022 Nov 21;17(11):e0277973. doi: 10.1371/journal.pone.0277973 (PMC9678311; doi:10.1371/journal.pone.0277973)
Supplement: S1 Table — Results shown as mean ± standard error of the mean. M/F: male/female; %M: percentage of males in the sample; BMI: body mass index; 6MWD: six-minute walking distance. P-value for comparison between ILD matched and unmatched population. (DOCX) [file pone.0277973.s001.docx]

**S1 Table**

|  | **ILD (n= 45)** | | | **ILD unmatched (n=3)** | | | **p-value** |
| --- | --- | --- | --- | --- | --- | --- | --- |
| Age | 66 | ± | 1 | 63 | ± | 7 | 0.666 |
| Gender M/F (%M) | 14/31 |  | (31%) | 3/0 |  | (100%) | **0.015** |
| BMI | 27.8 | ± | 0.7 | 22.0 | ± | 2.7 | **0.032** |
| 6MWD | 480 | ± | 18 | 434 | ± | 60 | 0.514 |
| Daily steps | 5631 | ± | 459 | 9264 | ± | 1653 | 0.113 |
| Daylight | 811 |  | 23 | 879 | ± | 53 | 0.463 |
