# Supplementary material for: Physical activity pattern of patients with interstitial lung disease compared to patients with COPD: A propensity-matched study
Source: PLoS One. 2022 Nov 21;17(11):e0277973. doi: 10.1371/journal.pone.0277973 (PMC9678311; doi:10.1371/journal.pone.0277973)
Supplement: S2 Table — IIP: idiopathic interstitial pneumonias; IPF: idiopathic pulmonary fibrosis; INSIP: idiopathic nonspecific interstitial pneumonia; COP: cryptogenic organizing pneumonia; IIP: idiopathic interstitial pneumonias; EAA: extrinsic allergic alveolitis; CTD-ILD: connective tissue disease-associated interstitial lung disease. (DOCX) [file pone.0277973.s002.docx]

**S2 Table**

| IIP   - IPF - INSIP - COP - IIP | 24 (53%)  17 (38%)  5 (11%)  1 (2%)  1 (2%) |
| --- | --- |
| Known causes   - EAA - Drug induced - Asbestosis - CTD-ILD | 19 (42%)  11 (25%)  1 (2%)  1 (2%)  6 (13%) |
| Unclassifiable ILD | 2 (4%) |
