# Supplementary material for: Physical activity pattern of patients with interstitial lung disease compared to patients with COPD: A propensity-matched study
Source: PLoS One. 2022 Nov 21;17(11):e0277973. doi: 10.1371/journal.pone.0277973 (PMC9678311; doi:10.1371/journal.pone.0277973)
Supplement: S3 Table — BMI: body mass index; FEV1: forced expiratory volume in the first second; FVC: forced vital capacity; DLCO: diffusion capacity for carbon monoxide; 6MWD: six-minute walking distance; QF: quadriceps force. r: Pearson correlation coefficient; p: p-value. (DOCX) [file pone.0277973.s003.docx]

**S3 Table**

|  |  | ILD | COPD | Healthy |
| --- | --- | --- | --- | --- |
| Age | r | -0.404 | -0.043 | -0.292 |
|  | p | **0.006** | 0.778 | 0.116 |
| BMI | r | 0.080 | -0.114 | 0.012 |
|  | p | 0.600 | 0.456 | 0.949 |
| FEV_1_, %pred | r | 0.261 | 0.524 | 0.100 |
|  | p | 0.083 | **0.001** | 0.598 |
| FVC, %pred | r | 0.170 | 0.256 | 0.110 |
|  | p | 0.265 | 0.089 | 0.564 |
| DLCO, %pred | r | 0.228 | 0.554 | 0.079 |
|  | p | 0.132 | **0.001** | 0.682 |
| 6MWD, m | r | 0.348 | 0.739 | 0.077 |
|  | p | **0.019** | **<.0001** | 0.685 |
| QF, Nm | r | 0.025 | 0.212 | -0.238 |
|  | p | 0.874 | 0.201 | 0.206 |
